# Enhanced Mechanophore Activation in Hydrogel Networks Driven by Swollen Network Pretension

**DOI:** 10.1021/acs.jpcb.5c02492

**Published:** 2025-08-28

**Authors:** Meytal Forer, Alessio Maselli, Yifan Liao, Adan Hijaze, Thekra Msarwe, Joshua M. Grolman

**Affiliations:** † Materials Science and Engineering Department, 26747 Technion-Israel Institute of Technology, Haifa 3200003, Israel; ‡ Nano Science and Nano Technology, Technion-Israel Institute of Technology, Haifa 3200003, Israel; § Department of Chemical Science, University of Napoli “Federico II”, Napoli 80126, Italy

## Abstract

There is often a discrepancy between the strain required to activate mechanophores and incorporation in bulk materials, inhibiting these force sensors from many practical, commercial, and biological uses. The difference is particularly pronounced for biomimetic networks such as viscoelastic hydrogels, in which the distribution of strain is unclear due to dissipation. Here, we show that the activation of spiropyran mechanophores in alginate networks is related to cross-linking characteristics by comparing ionic and covalent bonds. Through a simple shear force setup using syringes and Bernoulli’s principle, we observe higher activation of spiropyran in ionic gels, regardless of mechanical versus ultraviolet light stimulus. This may be predominantly driven by differences in network pretension due to swelling, as the ring-closing reaction of merocyanine to spiropyran was similarly affected. These insights shed new light on understanding force propagation in complex networks, leading to higher mechanophore sensitivities in biologically similar materials.

## Graphical abstract

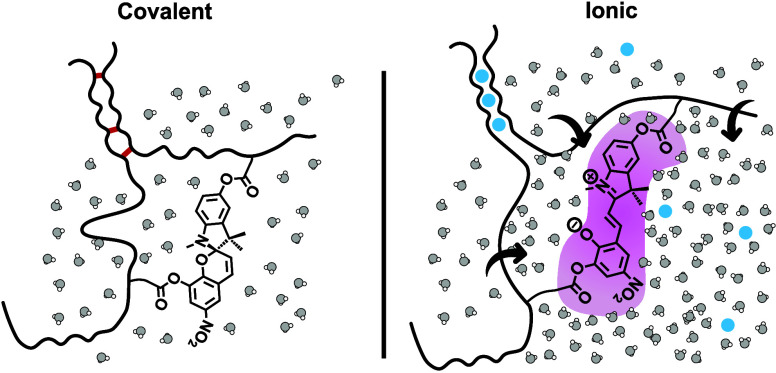

## Introduction

The desire to standardize measurements is not something new. Long ago, ancient civilizations in the Levant and the Nile Delta used *cubits* to measure building materials, agricultural goods, and volume.^1^ However, these measurements were related to the distance between a male’s elbow and middle finger.^2^ Despite the inconsistency of this quantification, it was sufficient to build complex societies and structures that still stand. Though we may take consistent measurements for granted today, they still pose major difficulties that inhibit the progress of science and engineering.

The challenges of standardization in measurement are similar for strain, where inconsistency may dominate due to material-specificity,^3^ lack of regio-spatiality,^4^ and cost.^5^ Although mechanophores often solve many of these issues,^6^ they are accompanied by several new ones. For one, they are quite sensitive in single-molecule force spectroscopy measurements^7^ or even through confined geometries simulate external force calculations (CoGEF), only requiring 240 pN of force to activate spiropyran (SP).^8^ However, once incorporated into an elastic bulk polymer, they often need an excess of 100% strain to activate.^9^ Recent work has continued to push the boundaries on activation sensitivity^10–12^ through electron-donating/withdrawing substituent groups along the ring structure^13^ or by biasing mechanophore placement in the center of polymer chains^14^ or at entanglement points.^15^ However, this really just touches the surface of sensitivities required for the adaptation of mechanophores into many commercial applications, let alone biological ones.

Biological materials face another set of challenges in that they are often viscoelastic and exhibit dissipation, further complicating the story. Much of the literature on mechanophores has focused on elastomers,^16–19^ mainly because they are easier to handle, but they also tend to be most sensitive to activation with regard to stress.^20^ This is due, in part, to the toughness of the materials and the fact that more strain is concentrated on the mechanophore instead of dissipating through the network via polymer rearrangement and breaking of cross-links.^21,22^

As a viscoelastic hydrogel network, alginate materials are an ideal model for biological ones because they exhibit similar behavior.^23^ Not only does the system mimic tissues like bone marrow in controlled environments,^24^ but it also allows for independent tuning of various mechanical properties,^25^ such as plasticity^26,27^ and viscoelasticity^28^ in a defined way. Though recent work has demonstrated the importance of hydration on network remodeling,^29^ including how covalent versus ionic bonds can drive stress-relaxation behavior through migration of water,^30^ it remains unclear how a molecular-level force sensor like mechanophores experiences these forces. Though mechanophores in hydrogels with covalent^48^ or ionic^49^ cross-linkers have previously been studied, a direct comparison of the two in the same polymer system has not been done. One would expect that ionic cross-linked hydrogels would dissipate more kinetic energy through disruption of physical interactions,^31,32^ but this has yet to be investigated. Other studies on mechanophores in hydrogels suggest that the swelling is sufficient for activation^50–52^ and that pretension can facilitate activation of mechanophores, although they did not specifically address viscoelastic systems.

In this work, we tested two systems of alginate hydrogels, one covalently cross-linked with glutaraldehyde and one ionically cross-linked with divalent calcium. The goal was to investigate the role of cross-link bonds and dissipation in macromolecular hydrogels on mechanophore response.

## Methods

### Preparing Hydrogels with Controlled Mechanical Properties

To first prepare the mechanophore-laden hydrogels, tetrazine-decorated alginate (ALG-Tetrazine) was synthesized as previously described,^25^ and the product was confirmed by ^1^H NMR (Figure S1),^33^ with SP added dropwise.^33^ Two types of alginate hydrogels were prepared: one with covalent cross-links and another with ionic cross-links (Figure S2). To prepare the covalent gel, an aqueous solution of sodium alginate 1.6% w/w (1.5 mL) was mixed with glutaraldehyde 25% w/w (0.08 mL) using two conjoined syringes, and the solution was poured between dialysis membranes (12 kDa) and two glass frits. A solution of 0.6 M HCl (1 mL) was added dropwise through the fritted plates, forming a uniform gel (Figure S3).^34^ FTIR was conducted to corroborate the formation of C–O and O–C–O covalent bonds as a result of the gelation reaction with glutaraldehyde (Figure S4).^35^

### DMA Measurements

Young’s modulus was measured via uniaxial compression via dynamic mechanical analysis (DMA, Anton Paar MCR 702). The samples were subjected to a linearly increasing stress over time at a rate of 5 um/s, and the stress and Young’s modulus were calculated accordingly (eq S1).

### Nanoindentation

Nanoindentation (Chiaro, Optics 11 nanoindenter For Life) was achieved by using a 49 μm radius spherical probe, a loading parameter of 1 μN, and an indentation speed of 5 μm/s over a 5 × 5 (200 μm spacing) array.

## Results and Discussion

### Preparing Hydrogels with Controlled Mechanical Properties

After synthesizing the gels, a preliminary test confirmed the functionality of SP in the gels, where exposure to 365 nm ultraviolet (UV) light induced a transition to merocyanine,^36^ resulting in the change of color to a bright violet color, measured by an increase in the Blue/Green color channel ratio (Table S1).

The Young’s moduli of the two hydrogels were then measured to ensure control over the stiffness of the gels and that the activation sensitivity of SP could be determined independent of bulk rigidity. Young’s modulus was measured via uniaxial compression to ensure uniform mechanical properties when comparing the two cross-linking modalities. In addition, the Young’s modulus of sodium alginate solution with the addition of 0.6 M HCl (1 mL) was measured to ensure that the gelation was caused predominantly by glutaraldehyde covalent cross-linking (Figure S5). The results of Figure 1a show nonsignificant differences between the ionic and covalent gels with moduli of around 7.8 and 8.9 kPa, respectively, via a one-way ANOVA test. This was also achieved while maintaining the same polymer loading fraction for both conditions at 1.6% w/w, as it is known that this can have a significant impact on both the mechanical properties of the hydrogel^37^ and mechanophore activation.^38^ The distribution of the compressive modulus for the ionic gels was inherently more polydisperse, which is commonly observed for calcium sulfate hydrogels.^39^

**Figure 1:**
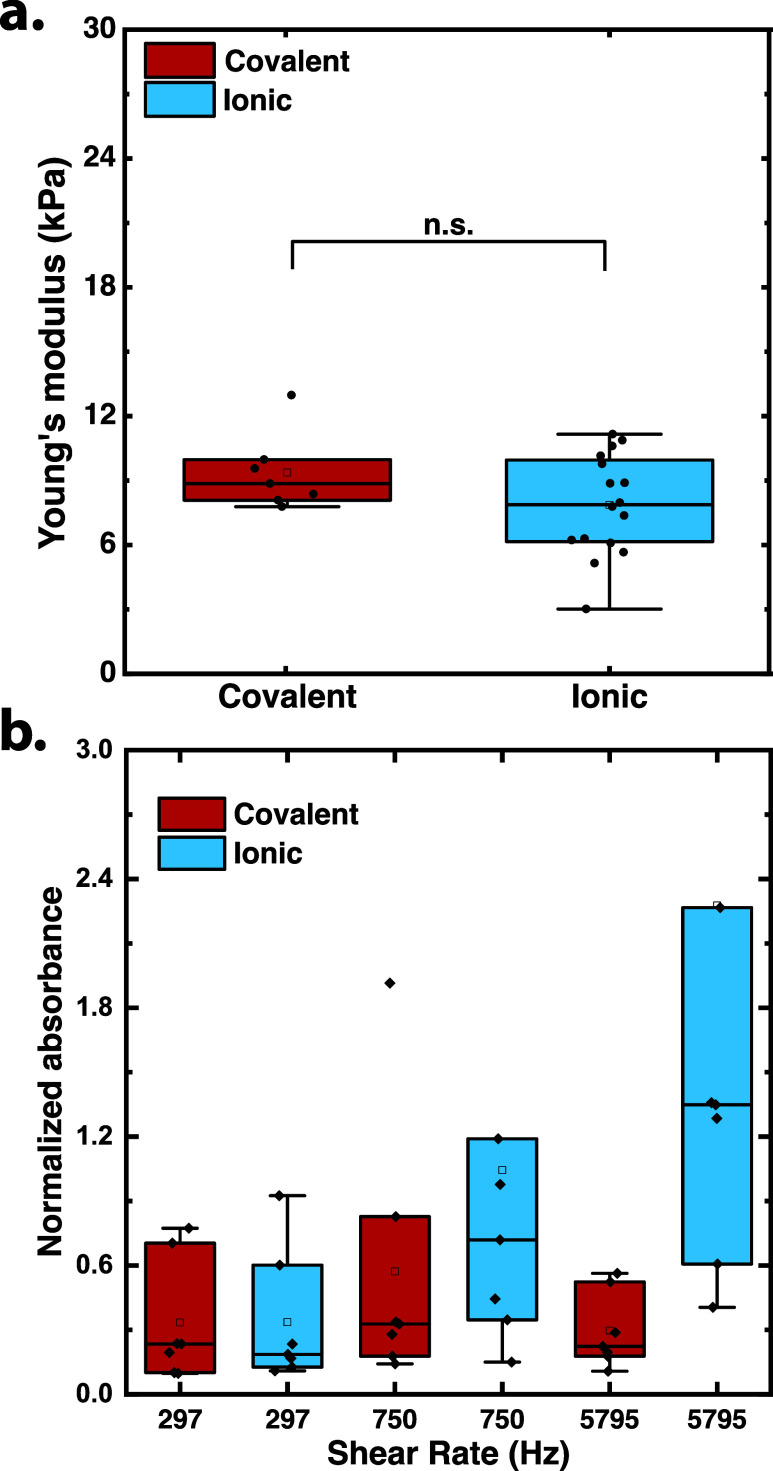
(a) Young’s modulus of covalent (*n* = 7) and ionic (*n* = 16) hydrogels measured on DMA; error bars are SD. (b) Absorbance of gels normalized by the mass of dry gels as a function of shear rate for covalent and ionic hydrogels (*n* = 7); error bars are SD.

### Shear-Stimulated Activation of Mechanophores through Needle Extrusion

The unique challenge of measuring mechanophore activation in hydrogels necessitated the development of a suitable technique to apply a substantial shear force on a relatively brittle macromolecular network. Previous experiments that have demonstrated the use of Bernoulli’s principle in flow systems required specialized equipment and focused on fully solvated polymers.^40^ Yet, these methods run the risk of clogging, especially for macromolecules, and do not probe shear rates that are biologically relevant. As the shear rate of capillaries in the human body ranges from several hundred to thousands Hz,^41^ we introduce a needle extrusion hydrogel system that can similarly cover such a range. By selecting needle gauges ranging from 15 to 25G, this spanned 297 to 5795 Hz. The shear rates were calculated (eq S2), indicating that decreasing needle size increased the shear stress applied (eq S3). The extruded gel was assumed to be isotropic, as orientation was not apparent in absorbance measurements.^53–55^ Subsequent extrusion was analyzed for absorbance at 560 nm (EPOCH 2 microplate reader), as the transition from SP to merocyanine (MC) is characteristic of the persistent conjugation past the spiro junction.^42,43^ The absorbance was dependent on both the thickness and concentration of the hydrogel samples (Figures S6–S8); therefore, the samples were all normalized by the dry mass after measurement. As shown in Figure 1b, the ionic cross-linked alginate hydrogels exhibited shear rate-dependent increases in absorbance, indicative of higher mechanical conversion of SP to MC. This was significantly higher than the covalent hydrogels, which did not show a similar rise in absorbance. This runs counter to our hypothesis that due to the lower dissipation in the covalent network,^44^ the strain would focus on the comparably weaker bonds of the SP mechanophore and therefore exhibit higher sensitivity. A higher variability in activation was also observed for the ionic gels due to the heterogeneous nature of the slurry cross-linking mechanism.

### Ca^2+^ Ions Do Not Significantly Affect SP Activation

The presence of Ca^2+^ ions may explain the enhanced sensitivity in the ionic hydrogels, since Ca^2+^ ions have been shown to lower the highest occupied molecular orbital (HOMO) and lowest unoccupied molecular orbital (LUMO) energy gaps of MC.^47^ It was demonstrated that in the presence of Ca^2+^ ions, the product HOMO energy of MC decreases by a few electron volts from −0.208 to −0.326 eV. Since the product has lower energy, there is a greater driving force for the transition from SP to MC in the presence of calcium ions.^47^ If calcium ions drove the differences in activation sensitivities between ionic and covalent, the absorbance of SP before and after activation by UV in aqueous conditions with the addition of CaSO_4_ should be different. Yet, as shown in Figure 2a, the absorbance of activated SP in Ca^2+^ solution is not substantially higher than that of activated SP in the absence of Ca^2+^. Moreover, in the macromolecular model, a sample of covalent gel was immersed in a CaSO_4_ solution (0.07M) to examine the effect of Ca^2+^ on hydrogel absorbance. A control measurement was immersed in the same concentration of Na_2_SO_4_ solution to keep the salt concentrations similar and avoid changes in the osmotic effects that might influence the swelling ratio in the network. The absorbance of the gel samples shown in Figure 2b suggests that the addition of Ca^2+^ did not affect the absorbance of covalent hydrogels by itself. If the absorbance of covalent gels did not change in the presence of Ca^2+^, then there may be another driver behind the observed differences in the MC conversion. The shift of the peak from 560 to 520 nm could be explained by the presence of tetrazine in the gels, which has been shown to be dominant.^33^ To explore other potential causes for the enhanced mechanophore activation in ionic hydrogels, we measured the pH to be approximately 5.9 for both sample types, indicating that it is also not likely to be a differentiating factor (Table S2). In addition, the recovery of SP in water solution and in CaSO_4_ solution was measured (Figure S9), demonstrating that Ca^2+^ cannot prevent the MC from recovering back to SP.

**Figure 2:**
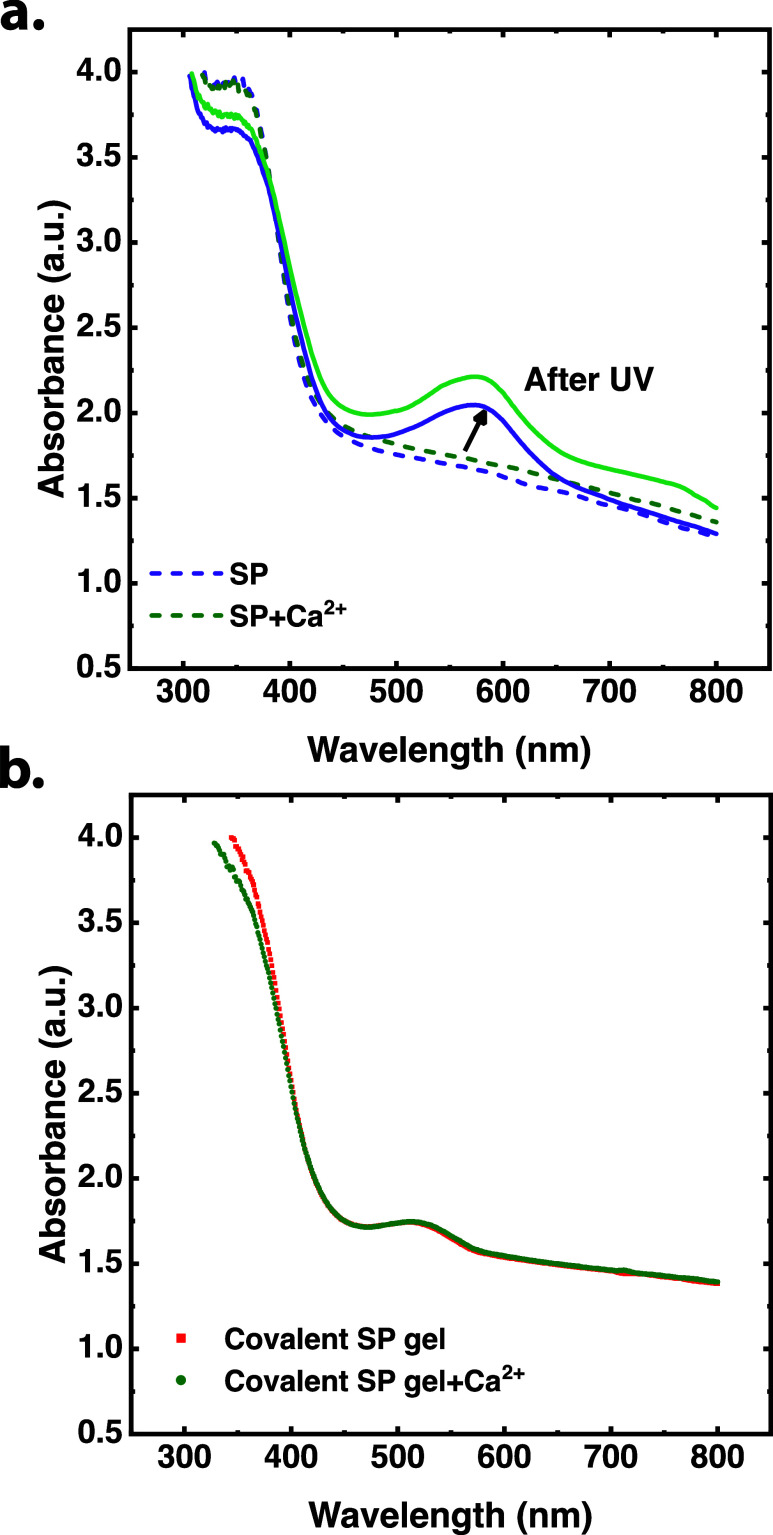
(a) Absorbance of SP in water solution and in CaSO_4_ solution, and absorbance of SP after activation to MC by using UV light under continuous exposure to Vilber Lourmat VL-6.LC 6W lamps in 365 nm mode for 10 min in distilled water solution and in CaSO_4_ solution. (b) Absorbance of covalent gels in Na_2_SO_4_ solution and in CaSO_4_ solution.

### Similar Recovery after Mechanical Activation, but Not after Photoactivation

To further probe the network structure, the SP in the hydrogel samples was activated by both UV light and mechanical shear force activation to ascertain any discrepancy between the method of activation and the recovery pathway. The idea being that if the recovery reaction of MC to SP was different as a result of the method of activation, this would suggest that the network remodeling occurred under shear stress. As UV activation and subsequent recovery are likely independent of any network conditions like pretension or swelling, the absorbency recovery should likely be negligible between ionic and covalent hydrogels.^45^

In Figure 3a, the absorbance of ionic and covalent gels was measured by activating the samples with UV light and after exposing the samples to white light for 24 h. It was noticed that the ionic hydrogel network recovered more than the covalent gel (Tables S3 and S4, and Figures S10 and S11). The recovery of the gels seemed to be unrelated to the dissipation of stress in the network and dependent on the initial structure. In Figure 3b, the absorbance of ionic and covalent gels was measured after applying mechanical force and after recovery in white light for 24 h. It can be seen that the recovery of both hydrogels was similar (Table S5). When the hydrogels were extruded from the syringe, the network structure may have changed due to water exclusion from the network, which has been demonstrated in similar alginate systems.^30^ Therefore, the importance of water and the role of network pretension deserve closer investigation.

**Figure 3:**
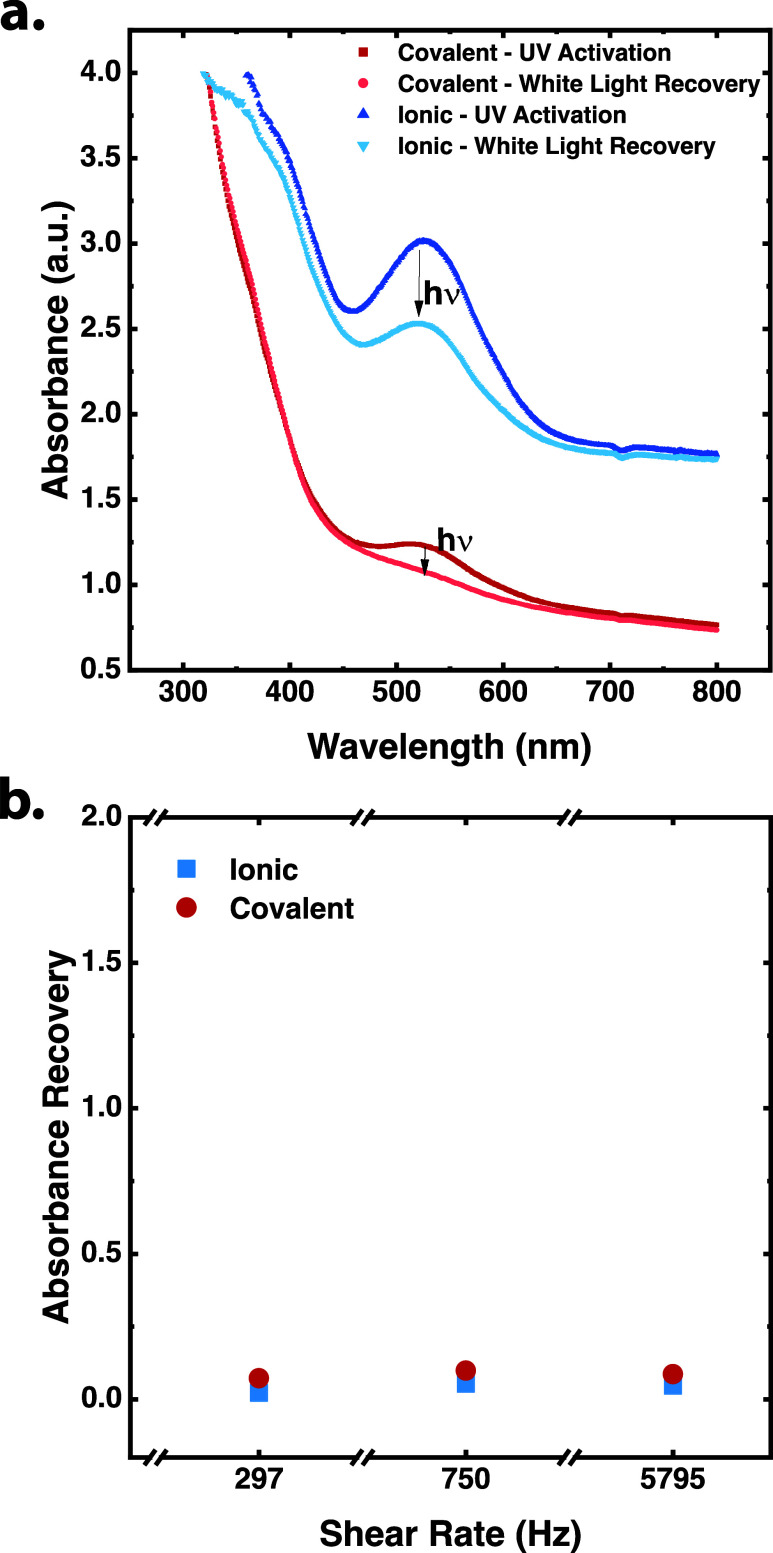
(a) Absorbance of covalent and ionic hydrogels after UV activation under continuous exposure to Vilber Lourmat VL-6. LC 6W lamps in 365 nm mode for 10 min and subsequent exposure to white light. (b) Absorbance of covalent and ionic hydrogels after mechanical stress and subsequent exposure to white light.

### Nanoindentation Reveals Differences in the Intrinsic Mechanical Properties of Hydrogels

The intrinsic properties of the two different hydrogel cross-linking systems may lead to differential activation of SP. Under bulk DMA measurements, they did not appear to be statistically different; however, it may be an issue of size scale, as the local heterogeneity of the networks might be a driver of activation sensitivity. To probe this, we turned to nanoindentation. From the data, the effective Young’s modulus was calculated (eq S4),^29^ and the results in Figure 4a show that ionic hydrogels were significantly stiffer compared to the covalent hydrogels. Even though their moduli were very close, with 5.1 kPa for the ionic and 4.3 kPa for the covalent on average, the statistical difference was substantial. It was also interesting to note that the distribution had roughly the same degree of heterogeneity, in that there may be more variation from batch to batch for ionic, but the local mechanical mapping of Young’s modulus was similar despite the cross-linking mechanism.

**Figure 4:**
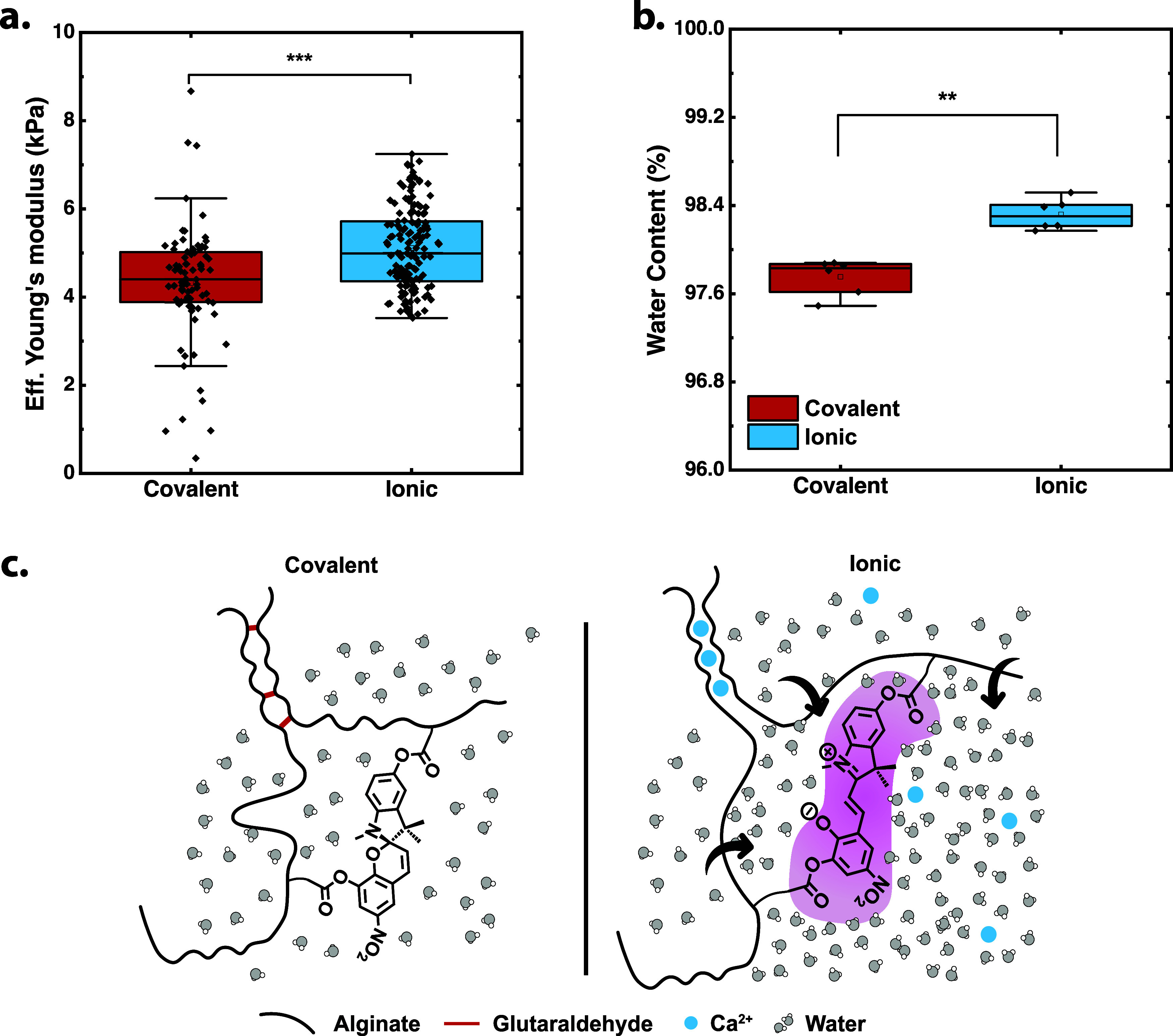
(a) Effective Young’s modulus measured via nanoindentation for covalent (*n* = 80) and ionic (*n* = 150) hydrogels; error bars are SD, *p* < 0.0005. (b) Water mass content of hydrogels measured on TGA (*n* = 6); error bars are SD, *p* < 0.005. (c) Schematic of pretension in ionic hydrogels compared to covalent hydrogels.

### Water Content Influences Network Pretension, Enhancing Mechanophore Response

Previous work has shown that water content can affect hydrogel stiffness through enlargement of alginate pores as measured by X-ray diffraction, leading to higher tension in the polymer chains.^29^ Similarly, in this case, even if the calcium ions may not be stimulating the activation through interaction with MC, they may draw with them a hydration shell of water 0.334 nm in hydrodynamic width^46^ and likely capable of swelling the hydrogel networks in a similar fashion. Using thermal gravimetric analysis (TGA; Figure S12 and eqs S6 and S7), we were able to demonstrate that not only are the ionic gels locally stiffer according to nanoindentation, but they also held significantly more water content at 98.3% per unit mass, compared to 97.7% (Figure 4b). In both cases, the tetrazine–alginate–SP conjugates were susceptible to slight changes in the salt concentrations (Figure S13). This phenomenon is described in Figure 4c, where we hypothesized that the enhanced water content and local Young’s modulus of the ionic gels were the result of a slightly more swollen network. In this case, there was greater polymer stretching and straightening, and the perceived activation sensitivity was higher in the bulk because less of the strain the network experiences is dissipated through polymer rearrangement. Moreover, the network structure likely rearranges in response to the shear field caused by Bernoulli principle extrusion, as supported by viscosity measurements (Figure S14). After such shear, the pretension in the ionic network may have been released, and the structure of ionic and covalent gels’ mechanophore activation and recovery responses became similar. While activation of SP within alginate networks under UV exposure should not alter the structure of the network, the recovery could be different between the ionic and covalent gels due to this differential pretension.

## Conclusions

In summary, this work demonstrates that even small differences in network hydration can have a significant impact on activation and recovery of mechanophores. Although the initial water amounts of both ionic and covalent hydrogels were equal, the small variances were sufficient to lead to differential activation sensitivities of embedded SP mechanophores. In the adaptation of mechanophores into more biologically relevant materials like hydrogels, understanding macromolecular factors that play a role in activation sensitivities becomes paramount.

## Supplementary Material



## Notes

Chemicals; gel preparation; ^1^H NMR; FTIR; Young’s modulus; shear rate; effective Young’s modulus calculations; absorbance normalization; and pH measurements (PDF)
