# Supplementary material for: Enhanced Mechanophore Activation in Hydrogel Networks Driven by Swollen Network Pretension
Source: J Phys Chem B. 2025 Aug 28;129(36):9249–55. doi: 10.1021/acs.jpcb.5c02492 (PMC12434674; doi:10.1021/acs.jpcb.5c02492)
Supplement: Supplementary file 1 [file jp5c02492_si_001.pdf]

Supporting information

Enhanced Mechanophore Activation in Hydrogel Networks Driven by Swollen Network Pretension

Meytal Forer, Alessio Maselli, Yifan Liao, Adan Hijaze, Thekra Msarwe, Joshua M. Grolman*

Materials Science and Engineering Department, Technion-Israel Institute of Technology, Haifa 3200003, IL.

Nano Science and Nano Engineering, Technion-Israel Institute of Technology, Haifa 3200003, IL.

Department of Chemical Science, University of Napoli "Federico II," Napoli, IT.

* Corresponding Author: JMG: grolman@technion.ac.il

Design and synthesis of force responsive sodium alginate gels

Alginate-tetrazine ^{1}H-NMR confirming the structure and tetrazine connection to the alginate chain[1]:

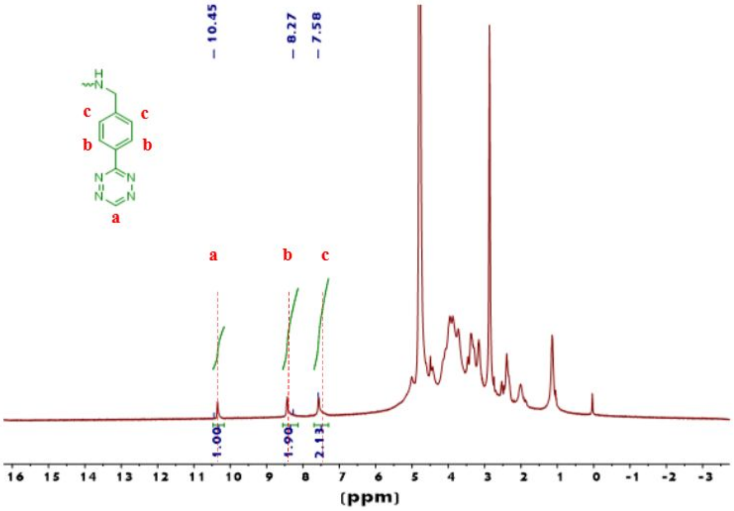

Figure S1: ^{1}H-NMR spectra of Alginate-tetrazine.

To prepare the ionic gel, an aqueous solution of sodium alginate 1.6% (w/w) was first prepared. Then, the solution was mixed with a calcium sulfate solution, which had been prepared by dissolving calcium sulfate (0.4 g) in water (40 mL). Using two syringes, the sodium alginate solution (1.5 mL) and calcium sulfate solutions (0.5 mL) were simultaneously combined. The

resulting mixture was then carefully poured between two glass plates, allowing for the formation of a uniform gel layer.

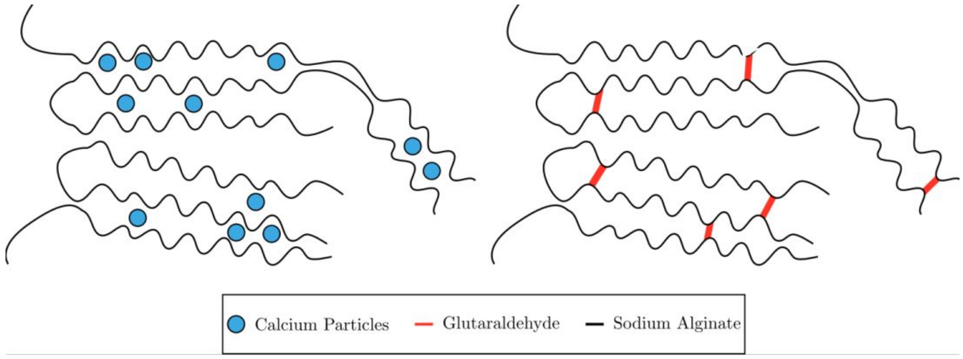

Figure S2: Schematic structure of ionic (left) and covalent (right) sodium alginate hydrogels.

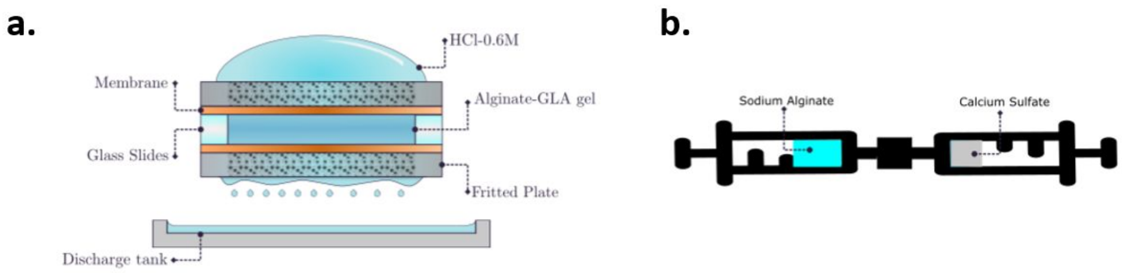

Figure S3: Schematic of synthesis hydrogels (a) covalent gel using fritted plates and (b) ionic gel using syringes.

FTIR

Attenuated total reflection-Fourier transform infrared (ATR-FTIR) spectra were recorded on a Thermo Scientific Nicolet FTIR iS50 spectrometer in the 400-4000 cm^{-1} range at room temperature. The FT-IR spectrum of sodium alginate with glutaraldehyde shows that in the 3600–1600 cm^{-1} region, three bands appear: a broad band centered at 3427.5 cm^{-1} assigned to hydrogen-bonded $\boldsymbol{v}_{O\text{-}H}$ band , the weak signal at 2927.0 cm^{-1} due to $\boldsymbol{v}_{C\text{-}H}$ band, and the $\boldsymbol{v(as)}_{O–C–O}$ assigned at 1615.6 cm^{-1}. The spectrum also exhibits a band at 1031 cm^{-1}, which was assigned to the $\boldsymbol{v}_{C–O}$.[2]

Increase in C-O Band Intensity:

A stronger C-O band after the reaction indicates the formation of acetals between glutaraldehyde

and sodium alginate. This peak is higher than in the sample without glutaraldehyde, indicating the formation of covalent bonds between glutaraldehyde and alginate.

Decrease in O-H Band:
A reduction in the O-H band intensity suggests the consumption of hydroxyl groups during the reaction.

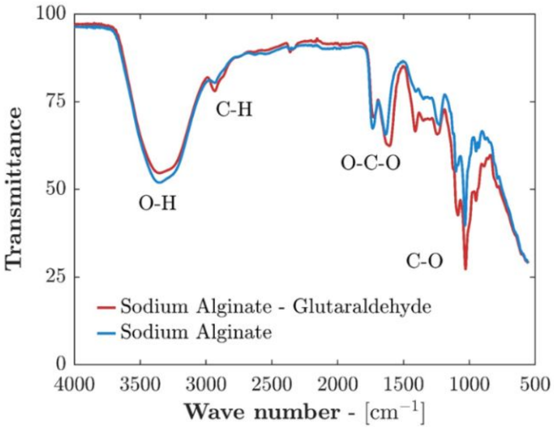

Figure S4: FTIR spectra of alginate and cross-linked alginate with glutaraldehyde.

UV measurements

A preliminary test confirmed the functionality of Spiropyran in the gels, samples was performed with the continuous light of Vilber Lourmat VL-6. LC 6W lamps in 365 nm mode for 10 minutes induced a transition to merocyanine, resulting in the change of color to a bright violet color, and increase in B/G ratio (**Table S1**).

Table S1: Activation of SP in alginate gels prior to mechanical activation to ensure functionality.

	**Covalent**		**Ionic**	
	Before activation	After activation in UV light	Before activation	After activation in UV light
B/G ratio	0.667	0.915	0.831	1.07
	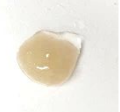	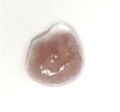	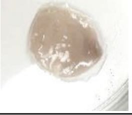	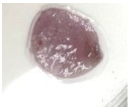

Hydrogels stiffness - Dynamic Mechanical Analysis

In addition to uniaxial compression in the DMA and measuring Young 's modulus of ionic and covalent hydrogels, Young's modulus of the alginate with only HCl was measured, to ensure that the main effect is related to the covalent cross-linker and not due to acidity.

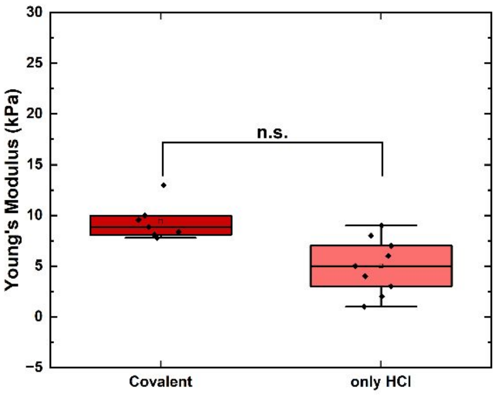

Figure S5: Young's modulus of Alginate gel with HCl only (n=9), and Covalent gel (n=7).

The sample was subjected to a linearly increasing pressure over time, and by measuring the change in force and displacement, the stress on the sample was calculated using the formula

$\sigma = F/A$, where F is the force applied, and A is the cross-sectional area of the sample. The strain was calculated using $\epsilon = \Delta L/L_i$, where ΔL is the change in the sample's height and L_i is the initial height. With the calculated strain and stress, the Young's modulus was determined using the equation (**Equation S1**):

$$\textbf{(S1)}\ E = \sigma / \epsilon$$

The calculations were done by the Anton-Parr software.

Mechanical activation by shear stress

The shear rates were calculated (**Equation S2**)

$$(\mathbf{S2})\ \gamma = \frac{4Q}{\pi r^3}$$

Where γ is the shear rate, Q is the volumetric flow rate (considering the volume squeezed out of the syringe and the time in seconds taking to press the syringe), and r is the radius of the needle.

For example, the volumetric flow when 0.2 mL of gel is squeezed in 2.5 seconds is $8*10^{-8}$ m^3/sec. The needle radius is 0.7 mm, and the shear rate when using the syringe 15G is calculated to be 297 Hz:

$$\gamma = \frac{4Q}{\pi r^3} = \frac{4 \cdot 8 \cdot 10^{-8}}{\pi \cdot 0.0007^3} = 297_{Hz}$$

The rest shear rates are calculated in the same way. For the 21G needle is 750 Hz, and for 25G is 5795 Hz.

The shear rate indicates the increasing shear stress which applied with the decreasing needle size (**Equation S3**)

$$(\mathbf{S3})\ \tau = \frac{4Q\eta}{\pi r^3} = \gamma \cdot \eta$$

Where τ is the shear stress, γ is the shear rate, and η is the viscosity.

Absorbance measurements

The UV-Vis absorbance spectra of both hydrogels after mechanical loading is shown here:

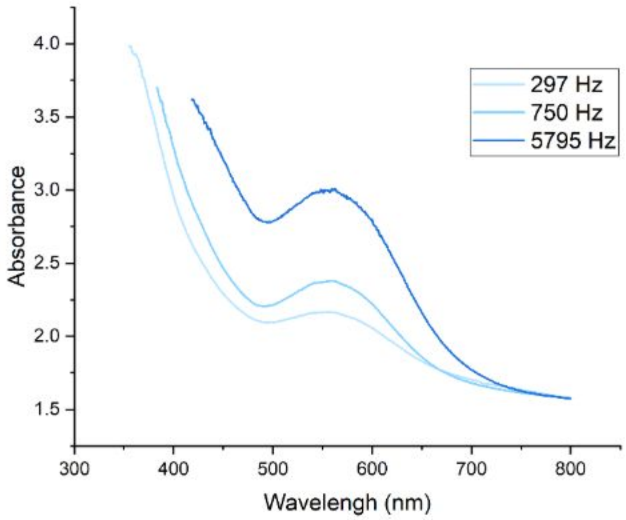

Figure S6: UV-Vis absorbance spectra of ionic hydrogel after mechanical loading.

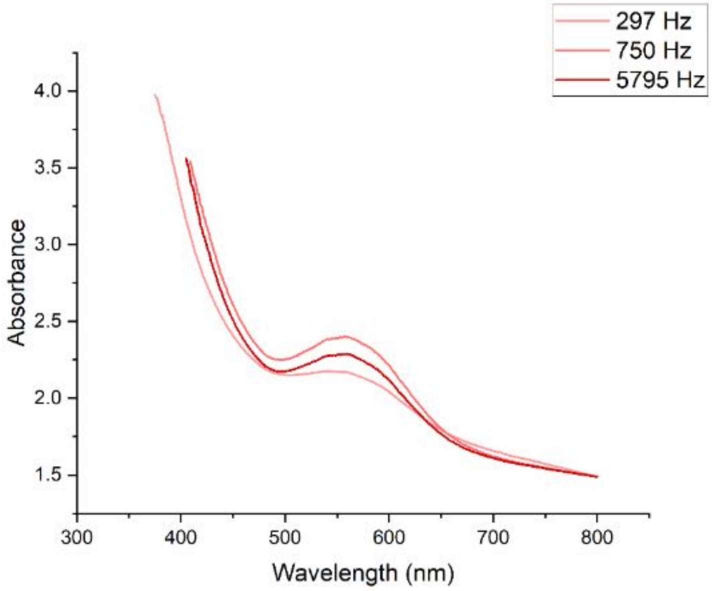

Figure S7: UV-Vis absorbance spectra of covalent hydrogel after mechanical loading.

The absorbance at 560 nm, were merocyanine absorbs[3] was divided by the mass of dry gel to normalize the results (**Figure S8**).

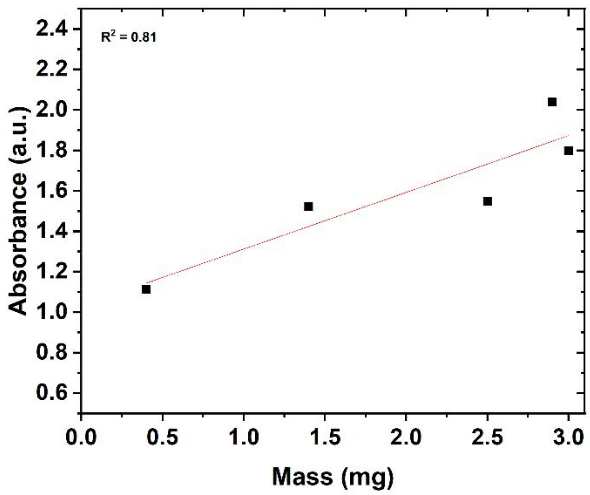

Figure S8: Absorbance of Alginate-tetrazine-SP gel as a function of dry gel mass.

Ca^{2+} ions effect

pH measurements

The pH was measured with pH meter to ensure that it doesn't affect the mechanophore sensitivity and is equal between ionic and covalent gels as shown in **Table S2:**

Table S2: pH of ionic and covalent alginate gels:

	**Ionic**	**Covalent**
pH	6	5.8

Recovery

The recovery of SP in water and in 0.07 M $CaSO_4$ solution was measured to ensure that the Ca^{2+} ions are not significantly affecting the recovery of SP.

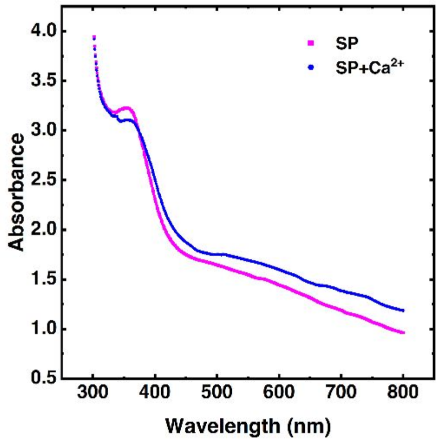

Figure S9: Absorbance of SP in water solution and in $CaSO_4$ solution after recovery.

Recovery of ionic and covalent gels after photoactivation was measured, and the percent of recovery was calculated by integrating the area under the curves, and conducting the recovered area from the activated curve.

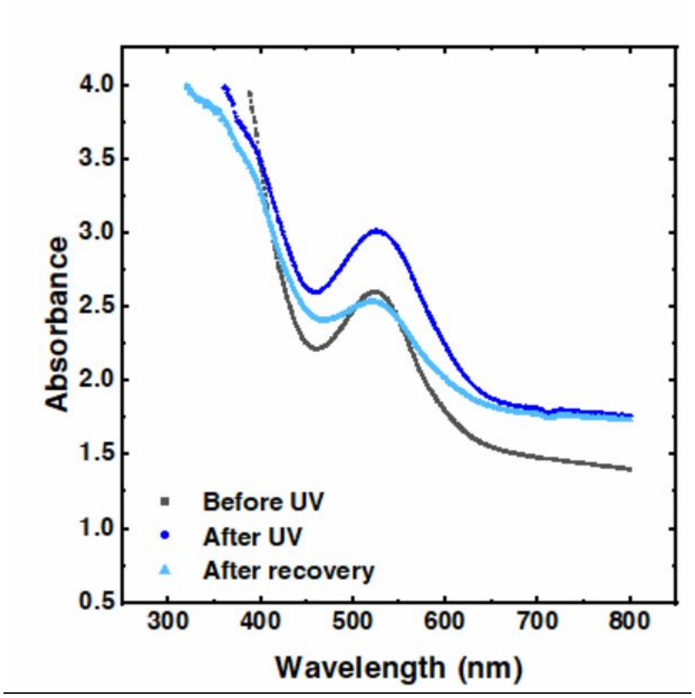

Figure S10: Absorbance of ionic hydrogel before, after UV activation and subsequent exposure to white light.

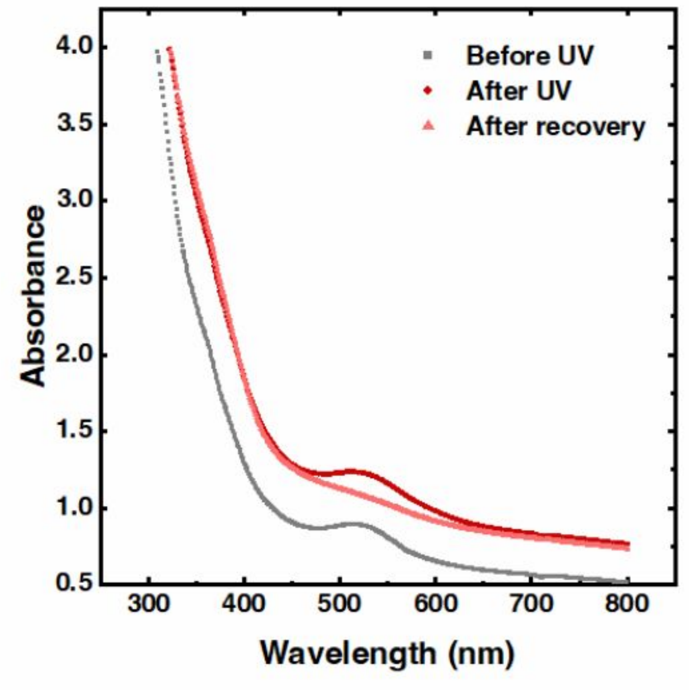

Figure S11: Absorbance of covalent hydrogel before, after UV activation and subsequent exposure to white light.

Table S3: area under the curves of covalent and ionic hydrogels before, after UV activation and subsequent exposure to white light:

Area under the curve	Ionic	Covalent
Before	747.154	501.121
After UV	895.988	639.326
After recovery	823.701	616.575

*Area under the curve was calculated by Origin software.

Table S4: Recovery (%) of ionic and covalent gels after photo activation:

Normalized area	Ionic	Covalent
After UV - before	148.834	138.205
After UV – after recovery	72.287	22.751
(After UV – after recovery)/(After UV - before)*100%	48.569%	16.462%

For mechanical activation -

Table S5: absorbance of ionic and covalent gel after mechanical activation and after recovery:

cross linker	Ionic			Covalent		
Shear rate	297	750	5795	297	750	5795
absorbance at 560 nm (after alignment) - after syringe squeeze	2.088	2.127	2.101	1.935	1.983	1.907
absorbance at 560 nm (after alignment) - after white light recovery	2.066	2.074	2.055	1.863	1.885	1.821
Absorbance recovery -distance before and after recovery	0.022	0.053	0.046	0.072	0.098	0.086

Nano indentation

Mechanical properties and stress stiffening phenomenon were quantified using an Optics 11 nano-indenter (Chiaro, Optics 11 For Life) and were run in a liquid environment (DDW). The nano-indenter was mounted with a 49 μm radius spherical probe made of Polystyrene, E = 6 GPa and ν = 0.325 and with a cantilever rigidity of 0.45 N/m. Young's modulus was obtained by fitting the force-displacement curve with a Ding linear elastic model (**Equation S4**), derived from the Hertz model (**Equation S5**) but applicable for larger deformations and hyperelastic materials[4]:

$$(\mathbf{S4})\ \ F = \frac{4}{3}E_{eff}\sqrt{R}h^{3/2}\cdot\left(1-0.15\frac{h}{R}\right)$$

$$(\mathbf{S5})\ E_{eff} = \frac{\frac{3}{4}F}{\sqrt{R}\cdot h^{3/2}}$$

Where, F is the measured force (N), E_{eff} the effective Young Modulus (Pa), R the radius of the indenting probe (m), and h is the indentation displacement (m).

We performed experiments and mapped the young's modulus along the gel. During indentations, location of the probe, displacement and force applied on the sample were recorded at a sampling rate of 1 kHz.

Young modulus calculations

Data was analyzed using Matlab (R2024a, MathWorks, USA). Contact point was automatically determined by the nano-indenter as a variation of 0.1 V signal variation corresponding to a displacement change about 5 nm from the baseline, and ν was considered equal to 0.5 due to the high presence of water composing the gel. Then the ding model was fitted on all the force displacement signal using nonlinear regression model function of Matlab.

TGA

Thermal Gravimetric Analysis (TGA) was conducted to measure water content in the gels. The samples were heated from 25℃ to 90℃ in heating rate of 10°C/min, then from 90-105℃ in heating rate of 2°C/min, and then from 105-170℃ in heating rate of 10°C/min. The mass loss precent was calculated for each step, and the total mass loss was calculated.

For example, one measurement of the covalent gels is presented here:

$$(\boldsymbol{S6})\ \ water\ content\ \% = 100 - 2.13 = 97.87\%$$

One measurement of the ionic gels is presented here:

$$(\boldsymbol{S7})\ \ water\ content\ \% = 100 - 1.48 = 98.52\%$$

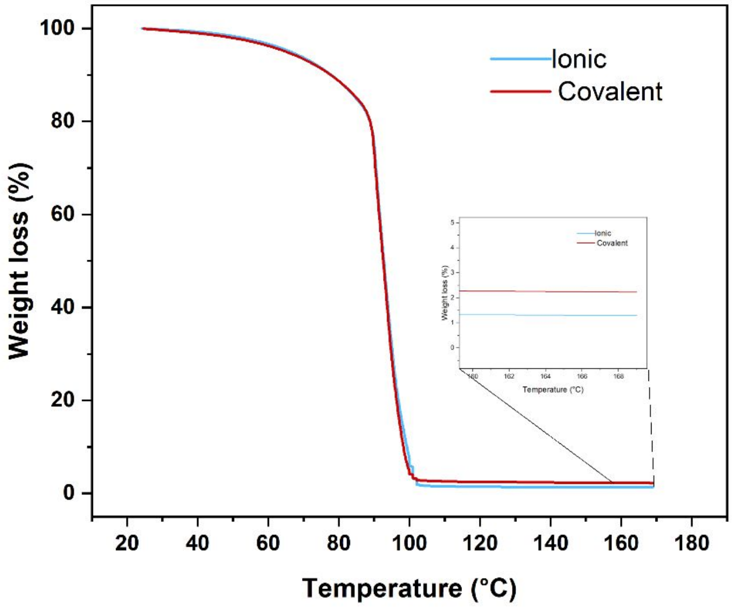

Figure S12: TGA spectra of covalent and ionic alginate gels measurement.

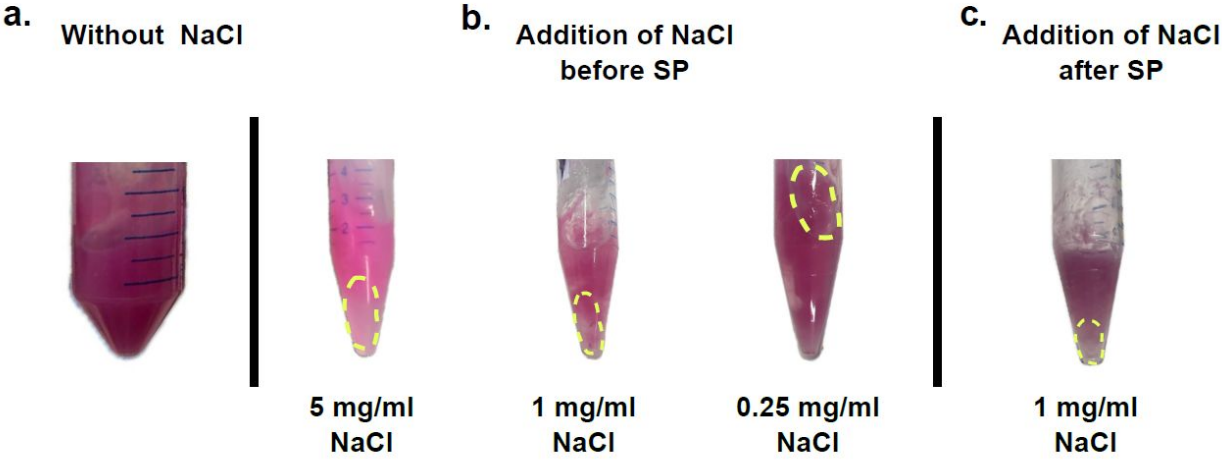

Figure S13: Precipitation of tetrazine-alginate and tetrazine-alginate-SP upon addition of NaCl at various concentrations

The viscosity of both hydrogels was also measured on the DMA, using parallel plates and shear stress VS. Shear rate was measured. The viscosity was calculated as the slope of the curve according to the next equation:

$$\textbf{(S8)}\ \eta = \frac{\tau}{\gamma}$$

Where τ is the shear stress, γ is the shear rate, and η is the viscosity.

The calculations were done by the Anton-Parr software.

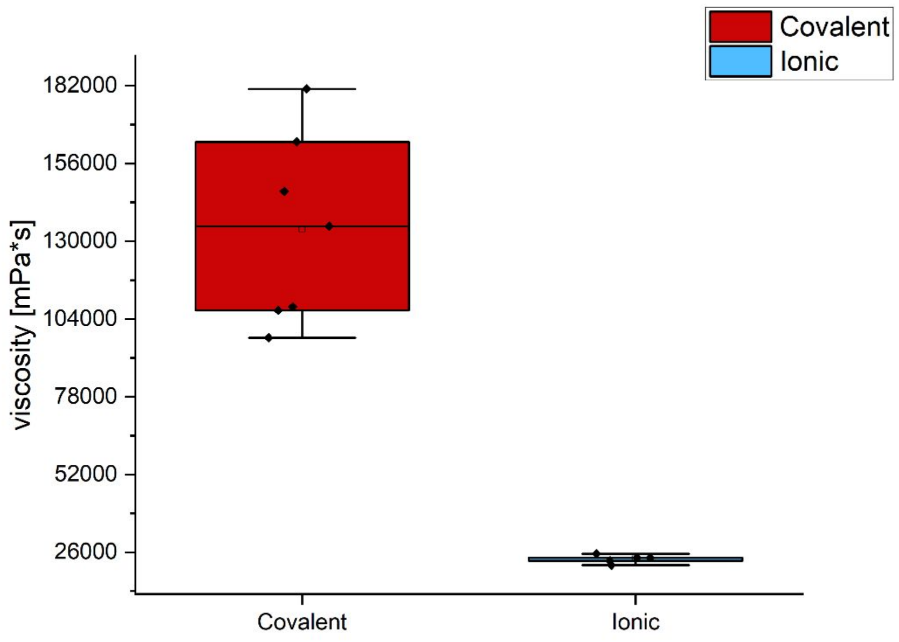

Figure S14: viscosity of covalent (n=7) and ionic (n=5) hydrogels measured on the DMA. Error bars are SD.

REFERNCES

(1) Liao, Y.; Le Roi, B.; Zhang, H.; Diesendruck, C. E.; Grolman, J. M. Facile Mechanophore Integration in Heterogeneous Biologically Derived Materials via "Dip-Conjugation." *J Am Chem Soc* **2024**, *146* (26), 17878–17886. https://doi.org/10.1021/jacs.4c03534.

(2) Leal, D.; Matsuhiro, B.; Rossi, M.; Caruso, F. FT-IR Spectra of Alginic Acid Block Fractions in Three Species of Brown Seaweeds. *Carbohydr Res* **2008**, *343* (2), 308–316. https://doi.org/10.1016/J.CARRES.2007.10.016.

(3) Kohl-Landgraf, J.; Braun, M.; Özçoban, C.; Gonçalves, D. P. N.; Heckel, A.; Wachtveitl, J. Ultrafast Dynamics of a Spiropyran in Water. *J Am Chem Soc* **2012**, *134* (34), 14070–14077. https://doi.org/10.1021/JA304395K.

(4) Le Roi, B.; Grolman, J. M. Hydration Effects Driving Network Remodeling in Hydrogels during Cyclic Loading. *ACS Macro Lett* **2025**, *14*, 176–181. https://doi.org/10.1021/ACSMACROLETT.4C00653/ASSET/IMAGES/LARGE/MZ4C00653_0004.JPEG.
